# Optimizing Multiple Analyte Injections in Surface Plasmon Resonance Biosensors with Analytes having Different Refractive Index Increments

**DOI:** 10.1038/srep15855

**Published:** 2015-10-30

**Authors:** Massinissa Si Mehand, Bala Srinivasan, Gregory De Crescenzo

**Affiliations:** 1 Department of Chemical Engineering, École Polytechnique de Montréal. P.O. Box 6079, Centre-ville Station, H3C 3A7 Montréal, Québec, Canada

## Abstract

Surface plasmon resonance-based biosensors have been successfully applied to the study of the interactions between macromolecules and small molecular weight compounds. In an effort to increase the throughput of these SPR-based experiments, we have already proposed to inject multiple compounds simultaneously over the same surface. When specifically applied to small molecular weight compounds, such a strategy would however require prior knowledge of the refractive index increment of each compound in order to correctly interpret the recorded signal. An additional experiment is typically required to obtain this information. In this manuscript, we show that through the introduction of an additional global parameter corresponding to the ratio of the saturating signals associated with each molecule, the kinetic parameters could be identified with similar confidence intervals without any other experimentation.

Within the last decades, Surface Plasmon Resonance (SPR)-based biosensors have evolved to become an almost indispensable tool for the rigorous investigation of the interactions occurring between biomolecules^1^,^2^. The applications SPR biosensors to drug screening has already been acknowledged^3^ and their ever-growing popularity in this area is most likely related to the high sensitivity the recent SPR biosensors have reached, the high degree of automation as well as the availability of robust data analysis software packages that ease subsequent data analysis^4^,^5^,^6^.

In order to satisfy the throughput capacity required for drug screening campaigns (e.g. for fragment libraries^7^), most of the latest SPR-based biosensor developments have been directed to provide researchers with tools combining the flexibility and information-rich measurements of SPR devices to the high-throughput capacity of standard *in vitro* assays such as ELISA^8^. In this endeavour, SPR biosensing platforms being able to inject one sample against hundreds of immobilized targets, or multiple samples on multiple surfaces are now available. However, in most cases, the throughput capacity of these platforms is detrimental to the confidence level one can get on the kinetic parameters: classical low throughput SPR instruments still remain the most appropriate tools for precise kinetic parameter determination. A classical experiment with these instruments consists of 5-to-10 analyte injections. Of interest, Ö’nell and Andersson have demonstrated that two injections, when adequately chosen, allow for kinetic parameter determination with similar confidence as that of resulting from classical experiments^9^. With that in mind, our group has then developed an iterative optimisation algorithm aiming to reduce experimental time and material consumption under the desired confidence on kinetic parameters^10^. Recently, we went one step further by introducing an experimentation strategy based on simultaneous injections of two analytes combined with numerical optimization^6^. We demonstrated that this method further improves the throughput of the SPR while reducing material consumption. In this approach, the interactions between analytes and a given ligand had been modelled by assuming that the amplitude of the signal corresponding to each analyte was proportional to the mass accumulation on the surface, as already assumed by others^11^,^12^,^13^,^14^. This assumption is valid when the refractive index increments (RII) of the analytes are identical. Such is the case for proteins, for which RII values are around 0.18–0.19 ml/g. However, this hypothesis may not hold true for heavily glycosylated proteins, carbohydrates or small molecular weight compounds, for which RII values differing by up to two folds have been observed^15^. As reported by Davis and Wilson (2000), the impact of these RII discrepancies upon SPR measurements can be taken into account by determining the RII of the compounds with a differential refractometer. In an SPR-based screening campaign, the determination of the RII of each individual drug, prior to multiple-analyte injections would however significantly slow down the screening process. In this paper, we demonstrate that this issue can be alleviated by the use of an additional global parameter (α). This parameter corresponds to the ratio of the saturation levels (*R*_*max*_) of the molecules under investigation and thus takes into account RII discrepancies. We here demonstrate that this strategy is efficient to determine kinetic parameters since deviations of less than 10% were obtained when compared to values deduced from a standard experimental procedure (injection of one analyte at a time) while completely avoiding the experimentation required to calculate the RII of each individual drug.

## Materials and Methods

### Materials

Experimental data sets were generated with a BIACORE T100 optical biosensor equipped with research-grade CM5 sensor chips (GE Healthcare, Baie d’Urfe, QC). HBS-EP buffer, acetate buffer and ethanolamine were purchased from GE Healthcare. N-ethyl-N’-(3-dimethylaminopropyl) carbodiimide (EDC), N-hydroxysuccinimide (NHS), carbonic anhydrase isozyme II (CAII) that had been purified from bovine erythrocytes, 4-carboxybenzenesulfonamide (CBS), sulfanilamide, 1,3-benzenedisulfonamide (BDS), Sulpiride, Furosemide, 5-dimethyl-amino-1-naphthalene-sulfonamide (DNSA), dimethyl sulfoxide (DMSO) and phosphate buffer saline (PBS, 10 mM, pH 7.4) were purchased from Sigma-Aldrich Canada Ltd (Oakville, ON).

### Biosensor surface preparation

Biosensor surface preparation (CAII and blank surfaces) were performed according to published protocols^16^. CAII surface was prepared at density of approximately 5000 RU. After CAII immobilization or blank surface generation, the system was extensively primed with running buffer (HBS-EP).

### BIACORE sample injections

#### Analyte and buffer preparations

Analytes and buffer were prepared according to protocol reported in Navratilova *et al.* (2007). HBS-EP with 3% of DMSO was used as a running buffer. All the analytes were dissolved in pure DMSO ([CBS] = 1.76 mM, [BDS] = 0.35 mM, [sulphanilamide] = 1.67 mM, [Sulpiride] = 9.19 mM, [Furosemide] = 8.4205 mM, [DNSA] = 1.97 mM). These stocks were diluted by mixing 30 μl with 970 μl of pure HPS-EP, resulting in concentrations of 52.98 μM, 10. 49 μM, 50.27 μM, 275.81 μM, 252.61 μM and 59.10 μM for each analyte, respectively with 3% of DMSO in order to match the amount of DMSO in the running buffer.

#### Single analyte injections

Prior to analyte injections, 3 prime procedures and buffer injections were performed to stabilize the baseline of the instrument. All injections were performed in duplicate at a flow rate of 100 μl/min with a data collection rate set at 10 Hz, at 18 °C. For preliminary classical kinetic experiments, CBS, BDS, sulphanilamide, sulpiride and furosemide samples were injected at concentrations comprised between 264.90 nM and 52.98 μM, 52.45 nM and 10.49 μM, 251.37 nM and 50.27 μM, 1.37 μM and 275.81 μM or 315.80 nM and 252.61 μM respectively, in addition to 6 buffer solutions (for double referencing purpose), for 60 s across both control and CAII surfaces, followed by a maximum of 350 s injection of HBS-EP running buffer at 18 °C. As complete dissociation was observed in each case, no regeneration procedure was performed, in agreement with previous reports^16^,^17^.

#### Multiple analyte injections

**Figure 1:**
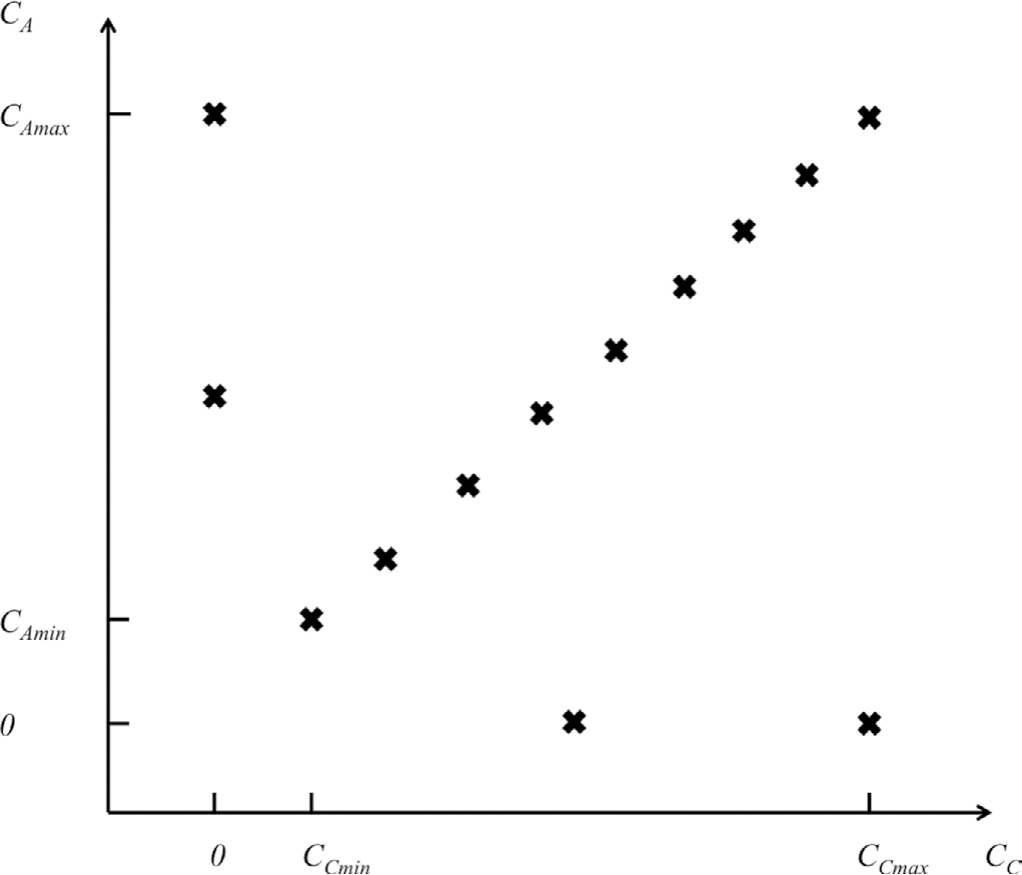
Experimental strategy for multiple-analyte injection experiments. For a given couple of molecules (denoted A and C), each cross corresponds to an injected mixture of A and C at final concentration indicated on the abscissa and ordinate, respectively. Minimal (i.e., *C*_*Amin*_, *C*_*Cmin*_) and maximal (i.e., *C*_*Amax*_, *C*_*Cmax*_) concentration values were identical to those used when injecting each drug individually (as indicated in the ‘Single analyte injections’ paragraph of the ‘Materials and Methods’ section).

Analytes were combined at different temperatures to create 11 case studies (including the case studies reported in^6^) as shown in Table 1. For each case study, 13 injections corresponding to various mixtures of drugs were chosen to cover the dimension space for concentrations (Fig. 1).

### Data analysis

In the case of classical injections (single-analyte injections), data sets were analyzed with Biacore T100 evaluation software, Biaevaluation 1.1.1, for kinetic parameter determination. For multiple-analyte injections and optimized-experiment analysis, in-house software packages were developed with the MATLAB 7.7.0.471 (R2008b) software platform (The Mathworks, Natick, USA). Those include the software package already described in the manuscript of Mehand *et al.* (2012) as well as the new multiple-analyte injection model that will be presented in detail in the subsequent sections. The optimization was solved with the standard simplex program available in the optimization Toolbox 4.1 of Matlab. In the case of multiple-analyte injections, for fast and sure convergence, the sensorgrams corresponding to the injection of single analytes at their highest concentrations ((*C*_*Amax*_, 0) and (0, *C*_*Cmax*_)) were fitted first with a simple Langmuirian model. The resulting parameter values were taken as a starting point to fit all the data.

## Results

### Signal-to-molecular weight study

**Figure 2:**
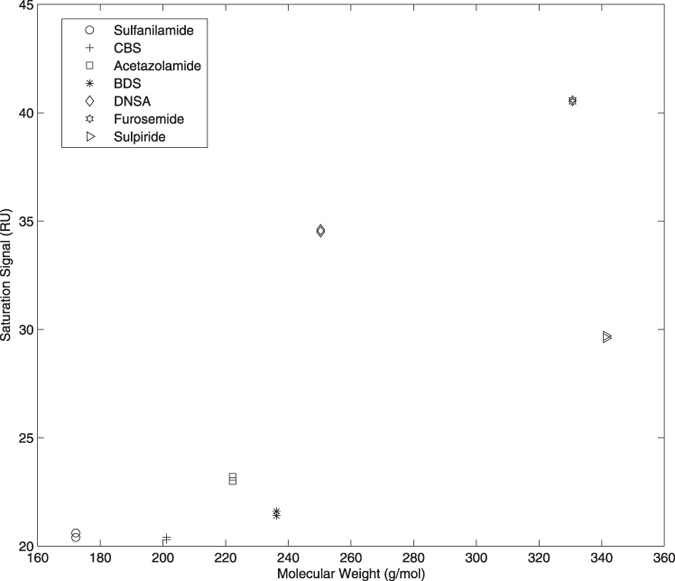
Observed saturation values (*R*_*max*_) as a function of the molecular weights of each analyte used in this study. *Rmax* values were determined on a single CAII surface (5000 RU) in duplicate.

In order to test extensively our multiple-analyte injection strategy while taking into account RII discrepancies, carbonic anhydrase isozyme II (CAII) was first immobilized at the surface of our SPR biosensor. CAII was selected since its interactions with many small molecular weight compounds have already been extensively characterized. Among known and well-characterized CAII binders, CBS, BDS, DNSA, sulphanilamide, sulpiride and furosemide were selected to cover a broad range of molecular weight (from 172 to 341.43 g/mol) and kinetic constants (Table 2). Each compound, known to interact according to a 1:1 stoichiometry with CAII, was injected at a concentration allowing to reach saturation at 18 °C, i.e., [CBS] = 52.98 μM, [BDS] = 10.49 μM, [Sulphanilamide] = 50.27 μM, [Sulpiride] = 275.81 μM, [Furosemide] = 252.61 μM, [DNSA] = 59.10 μM^16^,^17^,^18^,^19^. The plateau value of the resulting double-referenced sensorgrams corresponding to surface saturation was then plotted against their molecular weight in order to evaluate RII discrepancies. As can be seen in Fig. 2, there is no linear correlation between the molecular weight and the SPR signal for these small molecular compounds, thus indicating that most compounds had different RIIs. For example, CBS displayed saturation signals lower than sulphanilamide although sulphanilamide molecular weight is 14.51% lower than that of CBS. The same observation can be made for furosemide that gave a saturation level being lower than those of sulpiride and DNSA, in spite of their higher molecular weights.

In order to take into account these RIIs differences when combining multiple analytes, we thus proposed a new way to analyze sensorgrams resulting from multiple-analyte injections. In our approach, the ratio of the saturation levels of each analyte is estimated (α, global parameter) rather than experimentally determined with a differential refractometer, as previously proposed^15^. This model is described in the *Theory* section below.

### Theory

Assuming that both analytes A and C interact with the immobilized ligand (B) according to a simple 1:1 interaction model, their simultaneous injection results in the formation of two non-covalent complexes denoted AB and CB; the overall process being described by the following scheme:


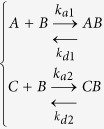


where *k*_a1_ and *k*_a2_ correspond to the related association rate constants (in *M*^*−1*^*s*^*−1*^), while *k*_d1_ and *k*_d2_ are the dissociation rate constants of the interactions (in *s*^*−1*^). The monitored signal thus corresponds to both ligand-analyte complexes (AB and CB) and the overall kinetics are described by the set of differential equations (1) and (2):


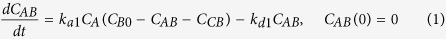



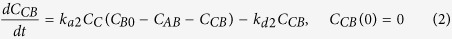


where *C*_*A*_ and *C*_*C*_ correspond to the concentrations of free analyte A and C, respectively; *C*_*B0*_ is the concentration of the ligand *B* at the beginning of the interaction, *C*_*AB*_ and *C*_*CB*_ are the concentration of the complexes AB and CB respectively (in *M*).


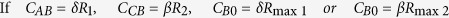


Then, equations (1) and (2) are equivalent to equations (3) to (5)


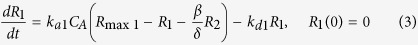



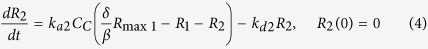



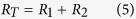


Since


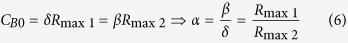


Where δ and β are proportionality constants related to analytes A and C respectively. These proportionality constants are used to translate AB and AC concentrations into corresponding SPR responses (*R*_*1*_ and *R*_*2*_, respectively). *R*_*T*_, *R*_*max1*_ and *R*_*max2*_ correspond to the recorded SPR signal over time and to saturating values for drug A and C, respectively.

### Model validation

Injections of various analyte pairs, i.e., (CBS, sulpiride), (CBS, furosemide), (BDS, sulpiride), (BDS, furosemide), (sulphanilamide, sulpiride), (sulphanilamide, furosemide) and (sulpiride, furosemide) were performed at 18 °C according to the experimental plan described in Fig. 1. Injection time was fixed at 60 s and the maximum dissociation time was 350 s. Each data set was control-corrected, double-referenced and used for parameter fitting. We also included in our analysis data we previously generated and published^6^. Those correspond to (CBS, sulphanilamide) injections at 18 °C, (BDS, sulfanilamide), (CBS, sulfanilamide) and (CBS, BDS) injections at 12°C (sensorgrams not shown in this manuscript). Three distinct models were used for comparison sake:

- Model #1: α is chosen equal to 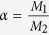 (as in Mehand *et al.*, 2012).
- Model #2: 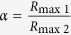; and R_max1_, R_max2_ are obtained from separate experiments (as in Davis and Wilson, 2000).
- Model #3: α is a free global parameter to be identified (our proposed method).

**Figure 3:**
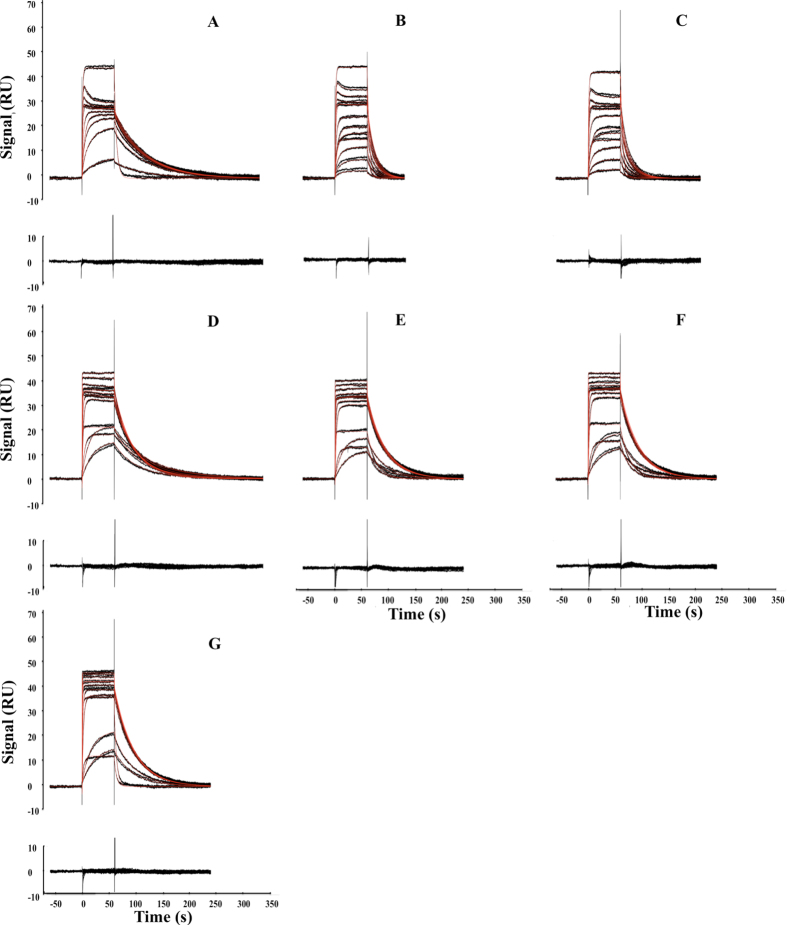
Kinetic analysis of multiple analyte injection experiments. Black dots correspond to control-corrected and double referenced sensorgrams for all case studies. Red lines correspond to global fits using model #3. (**A–G**) correspond to the injection of the following analyte pairs: CBS-sulpiride, BDS-sulpiride, sulfanilamide-sulpiride, CBS-furosemide, BDS-furosemide, sulfanilamide-furosemide and sulpiride-furosemide at 18 °C over CA II (5000 RU). The concentrations of CBS, BDS, sulphanilamide, sulpiride and furosemide were varied from 264 nM to 52.981 μM, 52 nM to 10.494 μM, 251 nM to 50.274 μM, 1.37  μM to 275.81 μM and 0.31 to 252.6 μM, respectively, according to the experimental strategy described in Fig. 1. For all sensorgram data set, related residual plot is given below.

The three models were chosen as the first corresponded to the one available in the manufacturer software packages, in which RII are considered equal. Data were also analyzed with Model #2, where the ratio of the RII was determined from separate experiments, as recommended by Davis and Wilson. To avoid the need of this additional experiment, the third model takes into account differences in RII values by introducing their ratio as a free parameter to be identified. Fits corresponding to Model #3 are presented in Fig. 3. In all cases, in order to increase the throughput of data analysis and mimic an automated data preparation routine, data were control-corrected without excluding any data point prior fitting (this explains the presence of the spikes at each injection transition in Fig. 3).

The deviations of the kinetic parameters determined with each model, from those determined using a classical single analyte injection strategy, are presented in Table 2. As expected, we observed the highest deviation of the kinetic parameters when applying Model #1 - These deviations reached more than 54% for several drug combinations and were correlated with bad fits as judged by visual inspection of the residual plots (difference between calculated and experimental data points; data no shown). Of salient interest, the maximal deviation decreased to 9% when Model #2 or Model #3 were used to analyze the data. The kinetic parameters identified with both models were similar. The fits related to the use of Model #3 were judged excellent (Fig. 3 and data not shown). Of interest, Model #3 was able to describe adequately the complexity of several sets of sensorgrams displaying transient maxima during the injection phase (Panels A, B and D of Fig. 3). This type of sensorgrams occurs when the analyte displaying the highest *R*_*max*_ value has faster kinetic rates than those of the other analyte.

Of salient interest, for each data set, it is possible to calculate an experimental α value (α_exp_, Table 1). The latter corresponds to the ratio of the plateau values (*R*_*max*_) corresponding to single analyte injections that were performed at *C*_*max*_, since these concentrations were known to correspond to saturation (see Fig. 1 for the experimental strategy). As can be seen in Table 1, these experimental values are in very good agreement with the values of α that were deduced from the global fit of the various sets of sensorgrams (α_fit_, Table 1).

## Discussion

In contrast to proteins, small molecular weight compounds are often characterized by non-identical RIIs. These discrepancies may translate into SPR signal amplitudes being different by up to two folds from those expected when taking into account their molecular weight and their stoichiometry of interaction with their bound partner^15^. The compounds we characterized in the present manuscript have different RIIs as deduced from the lack of linearity between the amplitudes of the SPR response at saturation and the analyte molecular weights (Fig. 2). While such a deviation from expected values does not affect the determination of the kinetic parameters when performing classical experiments (i.e., injections of a single analyte at various concentrations over a given surface), this RII difference must be taken into account for a valid kinetic analysis of multiple-analyte injections. As originally proposed by Davis and Wilson (2000), the experimental determination of the various RII and the use of these values to interpret the data would allow for a rigorous data analysis. However, such a procedure would be detrimental to throughput. In order to overcome this limitation, we here demonstrate that the introduction of a global parameter (α) corresponding to the ratio of the *R*_*max*_ values of the different analytes allowed for an adequate description of the mixed kinetics (Fig. 3) with deviations from kinetics obtained from a classical analysis of less than 10% (Table 1).

This result may appear at first counterintuitive as increasing the number of parameters may negatively impact their accurate identification. Let 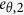 be the absolute error calculated from the estimation procedure corresponding to Model #2 and 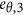 the absolute error calculated from Model #3. 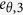 is typically larger than 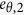 since the number of parameters are more. However, the value of α used in Model #2 is not known with certainty (due to error from the experiments that allowed its determination). The error in the parameter α of Model #2, 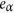, is computed by taking 0.3 RU error in both *R*_*max*_ values. This error propagates into the kinetic parameters and the propagated error in kinetic parameters with Model #2, 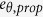, can be calculated using


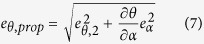


where 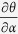 represents the sensitivity of the kinetic parameter estimates on this global parameter α. So, a decrease in confidence is caused in Model #3 by the increase of the number of parameters while in Model #2, it is due to error propagation. Calculations presented in Table 3, show that the confidence of these two approaches are similar. Thus, one could use the methodology corresponding to Model #3 with the same confidence as when determining RII experimentally, while saving experimental time and materials. In all the cases we studied here, the main assumption was that the analytes competed to bind to the same CAII site. The introduction of the α parameter could also be applied to other more complex kinetic models: for example, for the study of non-competitive or uncompetitive enzyme inhibition, were the enzyme might be the ligand while its substrate and inhibitor, the analytes.

We previously introduced the concept of multiple-analyte injections as a way to increase the throughput of SPR biosensor devices. In that endeavour, the approach was combined to an online optimization algorithm to limit the duration and the number of multiple-analyte injections^6^. With this method, the experimental time and material consumption were shown to be reduced drastically. Beside the experiment work reported in this manuscript, the addition of the α parameter in the kinetic model describing multiple-analyte injections was also tested with our online optimization approach; our results indicated that the throughput and the overall performances of the method were not affected. More specifically, the introduction of the α parameter did not significantly impact the performances of the online optimization algorithm since we observed the same gains in experimental time and material consumption while the levels of confidence on the kinetic parameters were similar to those reported in this study.

## Conclusion

The kinetic model available in the Biaevaluation software package has been applied to analyze sensorgrams corresponding to analyte heterogeneity^13^,^14^,^20^. In this model, it is hypothesized that the part of signal corresponding to a given analyte is proportional to its molecular weight. However, in agreement with previous study by Davis and Wilson (2000), this assumption does not hold true for small molecular weight compounds, in turn leading to biases in kinetic parameter values. While it was proposed to determine the RII ratio through additional experiments, it is shown here that the kinetic parameters could be obtained with similar confidence without additional experimentation (thereby saving time and material) by treating the ratio of the RII as a global parameter to be identified.

## Additional Information

**How to cite this article**: Mehand, M. S. *et al.* Optimizing Multiple Analyte Injections in Surface Plasmon Resonance Biosensors with Analytes having Different Refractive Index Increments. *Sci. Rep.* **5**, 15855; doi: 10.1038/srep15855 (2015).

## Figures and Tables

**Table 1:** Observed kinetic parameter deviation related to the use of the different models.

Experiment	Individual compound	Model #1 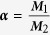	Model #2 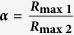	Model #3 Free parameter	α_fit_	α_exp_
CBS/Sulfanilamide (18 °C)	CBS	17.3%/7.6%	2.5%/3.4%	5.1%/0.03%	0.89	0.89
	Sulfanilamide	19.6%/15.2%	2.1%/1.3%	1.2%/1.5%		
BDS/Sulfanilamide (12 °C)	BDS	34.8%/19.2%	1.1%/1.8%	0.1%/2.4%	0.95	0.95
	Sulfanilamide	54.3%/13.6%	4.1%/1.1%	5.7%/1.6%		
CBS/Sulfanilamide (12 °C)	CBS	11.0%/1.3%	1.5%/2.8%	2.9%/0.6%	1.07	1.07
	Sulfanilamide	9.0%/29.5%	2.2%/5.8%	1.7%/7.8%		
CBS/BDS (12 °C)	CBS	17.0%/4.3%	4.5%/1.5%	5.5%/3.8%	1.02	1.03
	BDS	19.6%/8.6%	1.2%/2.5%	1.1%/2.0%		
CBS/Sulpiride (18 °C)	CBS	19.3%/0.8%	4.4%/0.8%	5.9%/0.3%	0.59	0.61
	Sulpiride	49.3%/5.1%	0.3%/4.5%	0.6%/5.2%		
BDS/Sulpiride (18 °C)	CBS	18.5%/2.0%	4.1%/3.8%	5.5%/5.1%	0.51	0.53
	Sulpiride	40.0%/2.9%	3.6%/5.6%	3.5%/6.8%		
Sulfanilamide/Sulpiride (18 °C)	Sulfanilamide	13.9%/6.1%	6.2%/7.2%	8.1%/8.0%	0.50	0.51
	Sulpiride	6.0%/4.1%	7.4%/4.2%	9.4%/6.7%		
CBS/Furosemide (18 °C)	CBS	21.8%/12.7%	7.2%/5.5%	5.0%/9.5%	0.52	0.51
	Furosemide	1.7%/8.5%	3.7%/8.1%	4.3%/9.6%		
BDS/Furosemide (18 °C)	BDS	49.5%/4.0%	7.5%/4.6%	6.1%/6.2%	0.54	0.55
	Furosemide	5.0%/10.6%	5.8%/7.4%	6.9%/8.9%		
Sulfanilamide/Furosemide (18 °C)	Sulfanilamide	43.1%/14.6%	7.3%/6.2%	8.9%/5.9%	0.57	0.58
	Furosemide	12.4%/9.4%	8.4%/9.1%	9.7%/9.2%		
Sulpiride/Furosemide (18 °C)	Sulpiride	28.2%/8.0%	1.2%/3.1%	0.3%/2.7%	1.14	1.13
	Furosemide	2.6%/2.9%	3.8%/2.7%	4.1%/3.1%		

For each scenario, observed deviations of the kinetic parameters (*k*_a_, *k*_d_) from those determined using a classical single-analyte injection approach, are given. α_fit_ corresponds to α, as deduced from the global fit of the various sets of sensorgrams. α_exp_ corresponds to the ratio of the plateau values (*R*_*max*_) corresponding to single-analyte injections that were performed at saturating concentrations.

**Table 2:** Kinetic parameters derived from single analyte experiments.

Temperature	12 °C		18 °C	
	k_a_ (×10^4^ M^−1^s^−1^)	k_d_ (×10^−3^ s^−1^)	k_a_ (×10^4^ M^−1^s^−1^)	k_d_ (×10^−3^ s^−1^)
BDS	7.86 ± 0.01	42.70 ± 0.04	10.87 ± 0.04	64.9 ± 0.3
CBS	2.23 ± 0.01	8.76 ± 0.02	2.75 ± 0.01	17.90 ± 0.04
Sulfanilamide	1.335 ± 0.005	34.3 ± 0.1	1.77 ± 0.01	68.7 ± 0.3
Sulpiride	–	–	0.26 ± 0.001	238 ± 2.5
Furosemide	–	–	3.90 ± 0.02	28.0 ± 0.2

**Table 3:** Calculated kinetic parameter confidences computed with Model #2 and Model #3.

Experiment	Individual compound	Model #2	Model #3
Sulfanilamide/Sulpiride (18°C)	Sulfanilamide	2.74%/1.56%	3.76%/2.18%
	Sulpiride	6.72%/3.15%	5.66%/4.54%

For a given scenario, deviations of the kinetic parameters (*k*_a_, *k*_d_) as calculated from error propagation for Model #2 and Model #3.
